# Telephone triage service use is associated with better outcomes among patients with cerebrovascular diseases: a propensity score analysis using population-based data

**DOI:** 10.3389/fpubh.2023.1175479

**Published:** 2023-06-07

**Authors:** Ryo Deguchi, Yusuke Katayama, Hoshi Himura, Tetsuro Nishimura, Yuko Nakagawa, Tetsuhisa Kitamura, Shunsuke Tai, Junya Tsujino, Takeshi Shimazu, Yasumitsu Mizobata

**Affiliations:** ^1^Department of Traumatology and Critical Care Medicine, Osaka Metropolitan Graduate School of Medicine, Osaka, Japan; ^2^Department of Traumatology and Acute Critical Medicine, Osaka University Graduate School of Medicine, Suita, Japan; ^3^Division of Environmental Medicine and Population Sciences, Department of Social and Environmental Medicine, Osaka University Graduate School of Medicine, Suita, Japan; ^4^Osaka Municipal Fire Department, Osaka, Japan; ^5^Osaka General Medical Center, Osaka, Japan

**Keywords:** telephone triage service, cerebrovascular disease, acute stroke, triage protocol, propensity score-matched analysis

## Abstract

**Introduction:**

The telephone triage service is an emergency medical system through which citizens consult telephone triage nurses regarding illness, and the nurses determine the urgency and need for an ambulance. Despite being introduced in several countries, its impact on emergency patients has not been reported. We aimed to determine the effect of the telephone triage service on the outcomes of hospitalized patients diagnosed with cerebrovascular disease upon arrival after being transported by an ambulance.

**Methods:**

This retrospective study included patients with cerebrovascular disease who were transported by ambulance between January 2016 and December 2019. The primary outcome was discharge to home by day 21 of hospitalization. A total of 344 patients who used the telephone triage service were propensity score-matched to 344 patients who directly called for an ambulance.

**Results:**

Telephone triage service use was associated with discharge to home by hospital day 21 (crude odd ratio: 1.8; 95% confidence interval: 1.3–2.4) and was not significantly associated with survival on hospital day 21 in multivariate regression analysis.

**Conclusion:**

The prognoses of cerebral infarction, intracerebral hemorrhage, and subarachnoid hemorrhage depend on the time from symptom onset to treatment. Telephone triage services may allow patients to receive treatment more rapidly than traditional ambulance requests, resulting in improved patient outcomes. The findings of this study suggest that the use of telephone triage services is associated with improved outcomes in patients with cerebrovascular disease and indicate that the costs for medical expenses and disability may be greatly reduced in an aging society.

## 1. Introduction

Telephone triage services help in the provision of necessary emergency medical services to patients by enabling an evaluation of the patients’ conditions over the telephone. Using information provided by the patient, nurses assess the patient’s condition and can dispatch an ambulance, suggest appropriate hospitals, or send a visiting physician. This necessary service has been introduced in several countries, including the United Kingdom and Australia (1, 2). In Japan, telephone triage services were introduced in Tokyo in 2007 and in Osaka in 2009 (3). A previous study reported that the telephone triage service in the Osaka Prefecture effectively triaged patients, dispatched ambulances, and suggested appropriate medical institutions (4). The annual number of emergency medical consultations in Osaka Prefecture was mostly more than 100,000 (5).

Patients who are deemed as emergency cases based on telephone triage services should be admitted directly to the emergency department upon arrival by ambulance (2). The relationship between the use of telephone triage services and patient outcomes is unclear, though the use of telephone triage services is associated with a lower proportion of unfavorable patient outcomes (6, 7). In addition, it remains unclear what diseases it is especially effective for (6, 8).

As telephone triage services encourage patients to visit a hospital immediately by performing emergency triage according to a protocol, it may be more effective for patients with cerebrovascular diseases than for patients with other diseases, since the time from symptom onset to treatment significantly affects the prognosis of patients with cerebrovascular diseases. However, whether telephone triage services are effective for patients with cerebrovascular diseases remains unclear. If these services positively affect the prognoses of these patients, their use may become more widespread, which would further improve patient outcomes.

Using propensity score matching, this study evaluated the effects of a telephone triage service on the outcomes of patients with cerebrovascular diseases who were transported by ambulance and hospitalized.

## 2. Methods

### 2.1. Study design

This retrospective study included hospitalized patients with cerebrovascular diseases (International Classification of Diseases 10th edition [ICD-10] code: I60.0-I69.8) transported by the Osaka Municipal Fire Department (OMFD) between January 1, 2016, and December 31, 2019. Patients who were transported in the same ambulance with other patients and those with missing data were excluded from the analysis. Anonymized data from the Osaka Emergency Information Research Intelligent Operation Network system (ORION), published in 2022, were used in this study (7). This manuscript was prepared according to the Strengthening the Reporting of Observational Studies in Epidemiology statement to assess the reporting of cohort and cross-sectional studies (9).

### 2.2. Study area

Osaka City is the largest metropolitan area in western Japan (225.33 km^2^), with a population of approximately 2.75 million people (10). A total of 94 medical institutions receives ambulances in Osaka City, and the total number of ambulance dispatches by the OMFD during the study period was 942,778 (11).

### 2.3. Telephone triage service in Osaka City

The telephone triage service in Osaka City has been described previously (4). In summary, a telephone triage nurse assesses the urgency of a caller’s symptoms using only the telephone triage protocol for each chief complaint (4). In principle, the emergency assessment in this service does not require doctors and is not affected by patients’ requests. This telephone triage service is similar to those in the United States, Canada, and the United Kingdom (1, 12, 13). Telephone triage services and ambulance requests in Japan are public services that are free of charge. Based on patient assessments, telephone triage nurses can dispatch an ambulance or suggest an appropriate medical institution (14). The Japanese telephone triage protocol includes 98 chief complaints for adults and children, and patients are triaged according to the signs and symptoms related to the chief complaints (14). In addition, data regarding telephone triage, such as sex, patient age, time of call initiation, end time of call, chief complaint, signs, urgency assessment, and whether an ambulance was dispatched or not, are recorded using the software. During the study period, 466,744 emergency medical consultations were conducted in the Osaka prefecture, including 20,387 ambulance dispatches requested by the telephone triage nurses (14).

### 2.4. Ethical consideration

This study was approved by the Institutional Review Board of Osaka Metropolitan University Hospital (approval number: 2021–233). As we used anonymized data provided by the OMFD, the requirement of obtaining patients’ informed consent was waived.

### 2.5. Main outcome

We defined the primary outcome of this study as the proportion of patients diagnosed with cerebrovascular disease upon arrival at the hospital by ambulance who were discharged to home on hospital day 21 (15). We wanted to consider neurological assessment as an outcome of cerebrovascular disease, but we did not have the relevant assessment information (such as Glasgow Outcome Scale and modified Rankin Scale). We assumed that home discharge outcomes were correlated with better neurological outcomes. That’s why we used discharge to home as the primary outcome. The secondary outcome was defined as survival on hospital day 21 in patients diagnosed with cerebrovascular disease upon arrival at the hospital by ambulance.

### 2.6. Propensity score matching

Patient propensity scores were calculated using a logistic regression model with nine variables that existed before the use of the telephone triage service or were indicative of the patient’s condition, including age, sex, calendar year, season, time, holiday (including weekends), accident location, consciousness, and administrative districts. Seasons were defined as spring from April to June, summer from July to September, autumn from October to December, and winter from January to March. The time was considered daytime from 09:00 to 17:59 and nighttime from 18:00 to 08:59. The accident locations were categorized as residences or elsewhere. Consciousness was classified using the Glasgow Coma Scale (GCS) (16) in the ambulance: severe impairment, 3–8 points; moderate impairment, 9–11 points; mild impairment, 12–13 points; and clear, 14–15 points. Administrative districts were classified into 24 areas as defined by Osaka City (17).

The effects of telephone triage service use on patient outcomes were evaluated. One-to-one pair matching was conducted by nearest-neighbor matching without replacement between patients for whom an ambulance was dispatched by the telephone triage service and for those whom an ambulance was dispatched without telephone triage, using a caliper width of 0.2 of the standard deviation of the logit of the propensity score. Covariate balances before and after matching were confirmed by comparing the standardized mean differences (SMD). An SMD < 10% was considered as a negligible imbalance between the two groups.

### 2.7. Statistical analysis

Propensity score matching, univariate and multiple logistic regression models, and regression models with propensity scores as covariates were conducted. The variables used in the calculation of the propensity score and telephone triage services were used in the multiple regression model (Forced entry method). All statistical analyses were performed using SPSS version 25.0 J (IBM Corp. Armonk, NY, United States). All statistical tests were two-tailed, and statistical significance was defined as *p* < 0.05.

## 3. Results

**Figure 1:**
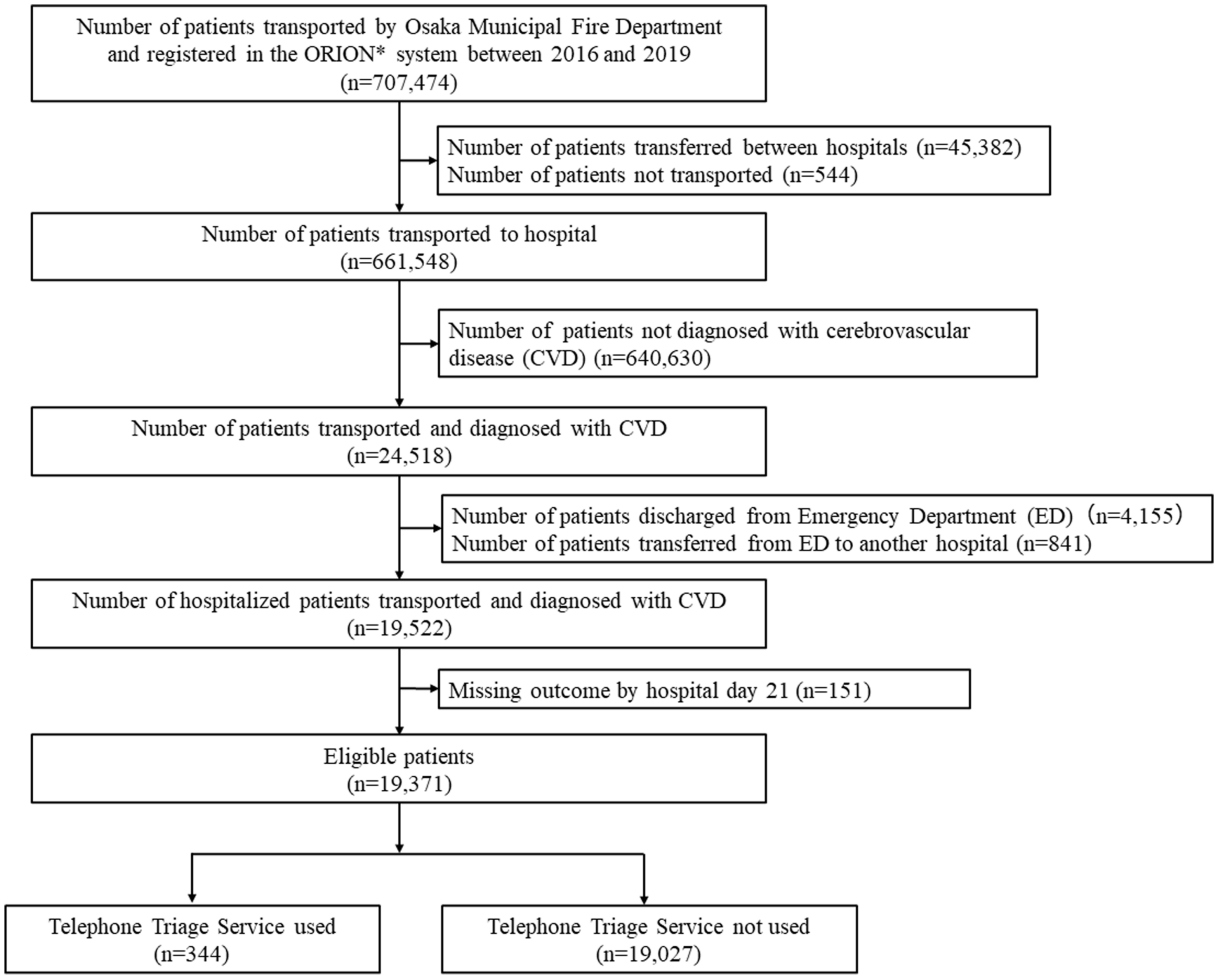
Patient flowchart in this study. Between 2016 and 2019, 707,474 patients were transported to medical institutions by Osaka Municipal Fire Department ambulances and were registered in the ORION system. A total of 45,382 patients who were transferred between hospitals and 544 patients who were not transported were excluded. Of the remaining patients, 24,518 had a diagnosis of cerebrovascular disease upon arrival at the hospital. To evaluate the inpatients’ outcomes, 4,155 patients who were discharged from the emergency department (including those who died), 841 patients who were transferred to another hospital, and 151 patients with missing data regarding hospital stay at day 21 were excluded. Ultimately, 19,371 patients were included in this study. Of these patients, 344 (1.8%) had used the telephone triage service and 19,027 (98.2%) had not. *ORION, Osaka Emergency Information Research Intelligent Operation Network.

Between 2016 and 2019, 707,474 patients were transported to medical institutions by OMFD ambulances and were registered in the ORION system (Figure 1). A total of 45,382 patients who were transferred between hospitals and 544 patients who were not transported were excluded from the study. Of the remaining patients, 24,518 had a diagnosis of cerebrovascular disease upon arrival at the hospital. To evaluate the inpatients’ outcomes, 4,155 patients who were discharged from the emergency department (including those who died), 841 patients who were transferred to another hospital, and 151 patients with missing data regarding hospital day 21 were excluded. Ultimately, 19,371 patients were included in this study. Of these patients, 344 (1.8%) had used the telephone triage service and 19,027 (98.2%) had not. Patients who used telephone triage services were younger and had a milder impairment of consciousness than those who did not (Table 1).

**Table 1:** Patient characteristics before and after propensity score matching.

	Telephone triage service used		Telephone triage service not used		SMD**	Telephone triage service used		Telephone triage service not used		SMD
	(*n* = 344)		(*n* = 19,027)			(*n* = 344)		(*n* = 344)		
Age (years)										
Mean (SD)	68.8	(13.9)	72.4	(13.8)	0.258	68.8	(13.9)	69.1	(14.2)	0.015
Sex										
Male	200	(58.1%)	11,026	(57.9%)	0.004	200	(58.1%)	208	(60.5%)	0.047
Female	144	(41.9%)	8,001	(42.1%)	0.004	144	(41.9%)	136	(39.5%)	0.047
Consciousness										
Severe impairment (GCS* 3–8 points)	10	(2.9%)	2,203	(11.6%)	0.339	10	(2.9%)	5	(1.5%)	0.100
Moderate impairment (GCS 9–11 points)	13	(3.8%)	2,150	(11.3%)	0.288	13	(3.8%)	20	(5.8%)	0.095
Mild impairment (GCS 12–13 points)	15	(4.4%)	1,432	(7.5%)	0.134	15	(4.4%)	13	(3.8%)	0.029
Clear (GCS 14–15 points)	306	(89.0%)	13,242	(69.6%)	0.492	306	(89.0%)	306	(89.0%)	0.000
Calendar Year										
2016	86	(25.0%)	4,592	(24.1%)	0.020	86	(25.0%)	93	(27.0%)	0.046
2017	94	(27.3%)	4,744	(24.9%)	0.054	94	(27.3%)	100	(29.1%)	0.039
2018	76	(22.1%)	4,832	(25.4%)	0.078	76	(22.1%)	76	(22.1%)	0.000
2019	88	(25.6%)	4,859	(25.5%)	0.001	88	(25.6%)	75	(21.8%)	0.089
Season										
Spring (from April to June)	79	(23.0%)	4,579	(24.1%)	0.026	79	(23.0%)	82	(23.8%)	0.021
Summer (from July to September)	86	(25.0%)	4,412	(23.2%)	0.042	86	(25.0%)	93	(27.0%)	0.046
Autumn (from October to December)	82	(23.8%)	5,088	(26.7%)	0.067	82	(23.8%)	75	(21.8%)	0.049
Winter (from January to March)	97	(28.2%)	4,948	(26.0%)	0.049	97	(28.2%)	94	(27.3%)	0.019
Time										
Daytime (9:00–17:59)	140	(40.7%)	10,235	(53.8%)	0.265	140	(40.7%)	135	(39.2%)	0.030
Nighttime (18:00–23:59, 0:00–8:59)	204	(59.3%)	8,792	(46.2%)	0.265	204	(59.3%)	209	(60.8%)	0.030
Day										
Weekday	189	(54.9%)	12,999	(68.3%)	0.278	189	(54.9%)	201	(58.4%)	0.070
Holiday including weekends	155	(45.1%)	6,028	(31.7%)	0.278	155	(45.1%)	143	(41.6%)	0.070
Accident location										
Residence	302	(87.8%)	11,955	(62.8%)	0.605	302	(87.8%)	305	(88.7%)	0.027
Elsewhere	42	(12.2%)	7,072	(37.2%)	0.605	42	(12.2%)	39	(11.3%)	0.027
Administrative district										
Kita-ku	14	(4.1%)	1,072	(5.6%)	0.073	14	(4.1%)	13	(3.8%)	0.015
Miyakojima-ku	19	(5.5%)	726	(3.8%)	0.081	19	(5.5%)	24	(7.0%)	0.060
Fukushima-ku	9	(2.6%)	404	(2.1%)	0.032	9	(2.6%)	9	(2.6%)	0.000
Konohana-ku	6	(1.7%)	494	(2.6%)	0.059	6	(1.7%)	9	(2.6%)	0.060
Chuo-ku	13	(3.8%)	970	(5.1%)	0.064	13	(3.8%)	17	(4.9%)	0.057
Nishi-ku	12	(3.5%)	457	(2.4%)	0.064	12	(3.5%)	11	(3.2%)	0.016
Minato-ku	15	(4.4%)	602	(3.2%)	0.063	15	(4.4%)	16	(4.7%)	0.014
Taisho-ku	7	(2.0%)	568	(3.0%)	0.061	7	(2.0%)	9	(2.6%)	0.039
Tennnoji-ku	13	(3.8%)	479	(2.5%)	0.072	13	(3.8%)	9	(2.6%)	0.066
Naniwa-ku	7	(2.0%)	511	(2.7%)	0.043	7	(2.0%)	8	(2.3%)	0.020
Nishiyodogawa-ku	13	(3.8%)	521	(2.7%)	0.059	13	(3.8%)	15	(4.4%)	0.029
Yodogawa-ku	15	(4.4%)	1,075	(5.7%)	0.059	15	(4.4%)	12	(3.5%)	0.045
Higashiyodogawa-ku	20	(5.8%)	1,112	(5.8%)	0.001	20	(5.8%)	16	(4.7%)	0.052
Higashinari-ku	19	(5.5%)	555	(2.9%)	0.130	19	(5.5%)	15	(4.4%)	0.054
Ikuno-ku	17	(4.9%)	955	(5.0%)	0.004	17	(4.9%)	15	(4.4%)	0.028
Asahi-ku	16	(4.7%)	627	(3.3%)	0.069	16	(4.7%)	13	(3.8%)	0.043
Joto-ku	14	(4.1%)	953	(5.0%)	0.045	14	(4.1%)	18	(5.2%)	0.055
Tsurumi-ku	9	(2.6%)	676	(3.6%)	0.054	9	(2.6%)	9	(2.6%)	0.000
Abeno-ku	13	(3.8%)	671	(3.5%)	0.013	13	(3.8%)	8	(2.3%)	0.085
Suminoe-ku	14	(4.1%)	869	(4.6%)	0.024	14	(4.1%)	13	(3.8%)	0.015
Sumiyoshi-ku	14	(4.1%)	983	(5.2%)	0.052	14	(4.1%)	16	(4.7%)	0.028
Higasisumiyoshi-ku	24	(7.0%)	948	(5.0%)	0.084	24	(7.0%)	26	(7.6%)	0.022
Hirano-ku	29	(8.4%)	1,353	(7.1%)	0.049	29	(8.4%)	31	(9.0%)	0.021
Nishinari-ku	12	(3.5%)	1,446	(7.6%)	0.180	12	(3.5%)	12	(3.5%)	0.000

GCS*, Glasgow Coma Scale; SMD**, Standardized Mean Differences.

Approximately 90% of telephone triage service users were located at their residences. A total of 344 patients from the group that did not use the telephone triage service were matched to those who used the service using propensity score matching, and the balance of each covariate improved between the two groups after propensity score matching. The area under the curve in the logistic regression model for propensity score calculation was 0.746.

The most common cerebrovascular events diagnosed among all patients were cerebral infarction (59.5%, 11,520 patients), intracerebral hemorrhage (25.2%, 4,880 patients), and subarachnoid hemorrhage (6.2%, 1,209 patients) (Table 2). Of the 19,371 patients included in this study, 7,551 (39.0%) were discharged home by hospital day 21, including 199 (57.8%) patients who used the telephone triage service and 7,352 (38.6%) patients who did not use the telephone triage service. Telephone triage service use was associated with discharge to home by hospital day 21 (crude odds ratio [OR]: 2.2; 95% confidence interval [CI]: 1.8–2.7), and was also independently associated with discharge to home by hospital day 21 (adjusted OR: 1.8; 95% CI: 1.5–2.3). This association was also observed in the propensity score-matched analysis (crude OR: 1.8; 95% CI: 1.3–2.4) (Table 3), when the propensity score was included as a covariate (adjusted OR: 1.8 95% CI: 1.4–2.2).

**Table 2:** Number of hospitalized patients with cerebrovascular diseases transported by ambulance in this study.

Diagnosis by ICD-10 code	Total (*n* = 19,371)		Telephone triage service used (*n* = 344)		Telephone triage service not used (*n* = 19,027)	
I60. Subarachnoid hemorrhage	1,209	(6.2%)	9	(2.6%)	1,200	(6.3%)
I61. Intracerebral hemorrhage	4,880	(25.2%)	55	(16.0%)	4,825	(25.4%)
I62. Other nontraumatic intracranial hemorrhage	698	(3.6%)	11	(3.2%)	687	(3.6%)
I63. Cerebral infarction	11,520	(59.5%)	254	(73.8%)	11,266	(59.2%)
I64. Stroke, not specified as hemorrhage or infarction	126	(0.7%)	1	(0.3%)	125	(0.7%)
I65. Occlusion and stenosis of precerebral arteries, not resulting in cerebral infarction	114	(0.6%)	2	(0.6%)	112	(0.6%)
I66. Occlusion and stenosis of cerebral arteries, not resulting in cerebral infarction	141	(0.7%)	1	(0.3%)	140	(0.7%)
I67. Other cerebrovascular diseases	366	(1.9%)	5	(1.5%)	361	(1.9%)
I68. Cerebrovascular disorders in diseases classified elsewhere	12	(0.1%)	0	(0.0%)	12	(0.1%)
I69. Sequelae of cerebrovascular disease	305	(1.6%)	6	(1.7%)	299	(1.6%)

**Table 3:** Discharge to home of hospitalized patients with cerebrovascular diseases transported by ambulance.

	Total		Telephone triage service used		Telephone triage service not used		Crude OR	(95% CI)	Adjusted OR	(95% CI)	*p* value
All patients	(*n* = 19,371)		(*n* = 344)		(*n* = 19,027)						
Discharge to home by hospital day 21	7,551	(39.0%)	199	(57.8%)	7,352	(38.6%)					
Univariate logistic regression model							2.2	(1.8–2.7)	–	–	<0.01
Multivariate logistic regression model*							–	-	1.8	(1.5–2.3)	<0.01
Regression model with propensity score as covariate							–	–	1.8	(1.4–2.2)	<0.01
Propensity score-matched patients	(*n* = 688)		(*n* = 344)		(*n* = 344)						
Discharge to home by hospital day 21	349	(50.7%)	199	(57.8%)	150	(43.6%)	1.8	(1.3–2.4)	–	–	<0.01

OR, odds ratio; CI, confidence interval. ORs were calculated for patients with versus without telephone triage service. *Adjusted for age, sex, calendar year, season, time, holiday including weekends, accident location, consciousness, and administrative district.

Of the 19,371 patients included in this study, 18,325 (94.6%) survived on hospital day 21, including 340 (98.8%) patients who used the telephone triage service and 17,985 (94.5%) patients who did not. Telephone triage service use was associated with survival on hospital day 21 by univariate regression model analysis (crude odds ratio (OR): 4.9; 95% confidence interval (CI): 1.8–13.2). However, telephone triage service use was not associated with survival on hospital day 21 by multivariate regression model analysis (adjusted OR: 2.4; 95% CI: 0.9–6.7). This association was also not observed when the propensity score was included as a covariate (adjusted OR: 2.0; 95% CI: 0.6–6.8) in the propensity score-matched analysis (crude OR: 2.0; 95% CI: 0.6–6.8) (Table 4).

**Table 4:** Survival of hospitalized patients with cerebrovascular diseases transported by ambulance.

	Total		Telephone triage service used		Telephone triage service not used		Crude OR	(95% CI)	Adjusted OR	(95% CI)	*p* value
All patients	(*n* = 19,371)		(*n* = 344)		(*n* = 19,027)						
Survival on hospital day 21	18,325	(94.6%)	340	(98.8%)	17,985	(94.5%)					
Univariate logistic regression model							4.9	(1.8–13.2)	–	–	<0.01
Multivariate logistic regression model*							–	–	2.4	(0.9–6.7)	0.09
Regression model with propensity score as covariate							–	–	2.0	(0.6–6.8)	0.27
Propensity score-matched patients	(*n* = 688)		(*n* = 344)		(*n* = 344)						
Survival on hospital day 21	676	(98.3%)	340	(98.8%)	336	(97.7%)	2.0	(0.6–6.8)	–	–	0.38

OR, odds ratio; CI, confidence interval. ORs were calculated for patients with versus without telephone triage service. *Adjusted for age, sex, calendar year, season, time, holiday including weekends, accident location, consciousness, and administrative district.

## 4. Discussion

The effects of telephone triage services on emergency care for cerebrovascular diseases in the urban areas of Japan were evaluated in this study. The most common cerebrovascular diagnosis was cerebral infarction, followed by intracerebral hemorrhage and subarachnoid hemorrhage. Among patients hospitalized with cerebrovascular diseases, telephone triage service use was associated with discharge to home by hospital day 21, which meant this service might be associated with improved neurological outcome (18). No significant associations were found between using this service and patient survival.

Previous studies have not reported the diagnoses of the patients who used telephone triage services. Therefore, it remained unclear what diseases the telephone triage service was effective for. The ORION system data includes ICD-10 coded diagnoses of patients transported by ambulance. In this study, the positive effects of telephone triage services on the outcomes of patients with cerebrovascular diseases were clarified. The prognoses of cerebral infarction, intracerebral hemorrhage, and subarachnoid hemorrhage are dependent on the time from symptom onset to treatment (19–21). Some treatments for these conditions cannot be implemented after a specific number of hours have passed from the time of symptom onset (22). Telephone triage service nurses may allow patients to receive treatment more rapidly than traditional ambulance requests, which may result in improved patient outcomes and lower medical and social security costs. While these findings may be applied worldwide, health care systems and ambulance request fees vary in different countries, which may affect the use and cost of telephone triage services.

The American Heart Association (AHA) describes the process from the onset of stroke to hospitalization as the ‘Ds’ of stroke care (23). The use of telephone triage services may shorten the detection (rapid recognition of stroke symptoms), dispatch (early activation and dispatch of emergency medical services system by calling 9–1-1), and delivery (rapid emergency medical service identification, management, and transport) processes described by the AHA. Citizens may choose to use telephone triage services that are free to the public over direct ambulance dispatch services due to the high costs associated with such services. The telephone triage service in the Osaka Prefecture used the face, arm, speech, time (FAST) acronym when triaging patients with stroke, which may have resulted in rapid and accurate ambulance dispatches. The FAST acronym is a simple screening algorithm created based on the Cincinnati Prehospital Stroke Scale (24). The algorithm identifies 88.9% of patients with stroke or transient ischemic attack and 99.9% of patients at acute onset (25). The use of telephone triage services may also shorten the time required to identify appropriate hospitals, improving the transportation process. Acute stroke is associated with a high risk of death and severe complications and requires long-term hospitalization, especially in older adults (26), and cerebral infarction is the most common disease among all hospitalized patients who arrive by ambulance (4). The findings of this study suggest that the use of telephone triage services is associated with improved outcomes in patients with cerebrovascular disease and indicates that the costs for medical expenses and disability may be greatly reduced in an aging society.

This study has several limitations. First, this study tended to include patients with mild cerebrovascular diseases. It remains unclear whether telephone triage will be useful for all severities of cerebrovascular diseases in ambulance transports. Telephone triage services may be unsuitable for patients with severe cerebrovascular diseases because it is reasonable to call an ambulance directly in such cases. Second, the outcomes of cases for which no ambulance was dispatched remain unknown. In the future, we plan to examine patients with cerebrovascular diseases for whom an ambulance was not called because of the telephone triage assessments. Third, we could not collect information related to cerebrovascular disease in this research, such as past medical history, or oral medication history. Fourth, the primary outcome of this study was discharge to home, which predicted the patients’ neurological outcomes, although it was affected by environmental factors including economic factors, such as income, and social factors, such as family composition. We were not able to adjust for these factors in this study; however, previous studies have shown that regional differences affect the outcomes of patients transported by ambulances (27), and we adjusted for this by including region-related factors as variables in the propensity score analysis. Finally, as this was an observational study, there are likely unknown confounding factors.

## 5. Conclusion

The use of telephone triage services is associated with improved outcomes among patients with cerebrovascular disease who are transported to hospitals by ambulance. These improved outcomes will likely reduce medical costs in an aging society.

## Data availability statement

The original contributions presented in the study are included in the article/supplementary material, further inquiries can be directed to the corresponding author.

## Ethics statement

The studies involving human participants were reviewed and approved by This study was approved by the Institutional Review Board of Osaka Metropolitan University Hospital (approval number: 2021–233). Written informed consent from the participants’ legal guardian/next of kin was not required to participate in this study in accordance with the national legislation and the institutional requirements.

## Author contributions

YK and TS conceived the idea of this study. ST and JT contributed to the data collection. RD, HH, YN, and YK contributed to the data analysis and interpretation. RD was a major contributor in writing the manuscript. YK, TN, TK, TS, and YM supervised the conduct of this study. All authors contributed to the article and approved the submitted version.

## Funding

This study was supported by the Fire and Disaster Prevention Technologies Program under Grant number JPJ000255.

## Conflict of interest

The authors declare that the research was conducted in the absence of any commercial or financial relationships that could be construed as a potential conflict of interest.

## Publisher’s note

All claims expressed in this article are solely those of the authors and do not necessarily represent those of their affiliated organizations, or those of the publisher, the editors and the reviewers. Any product that may be evaluated in this article, or claim that may be made by its manufacturer, is not guaranteed or endorsed by the publisher.
